# Rapid and Accurate Detection of Bacteriophage Activity against *Escherichia coli* O157:H7 by Propidium Monoazide Real-Time PCR

**DOI:** 10.1155/2014/319351

**Published:** 2014-11-02

**Authors:** Hui Liu, Yan D. Niu, Jinquan Li, Kim Stanford, Tim A. McAllister

**Affiliations:** ^1^College of Animal Science, Inner Mongolia Agricultural University, Hohhot, Inner Mongolia 010018, China; ^2^Lethbridge Research Centre, Agriculture and Agri-Food Canada, Lethbridge, AB, Canada T1J 4B1; ^3^Alberta Agriculture and Rural Development, Agriculture Centre, Lethbridge, AB, Canada T1J 4V6

## Abstract

Conventional methods to determine the efficacy of bacteriophage (phage) for biocontrol of *E. coli* require several days, due to the need to culture bacteria. Furthermore, cell surface-attached phage particles may lyse bacterial cells during experiments, leading to an overestimation of phage activity. DNA-based real-time quantitative polymerase chain reaction (qPCR) is a fast, sensitive, and highly specific means of enumerating pathogens. However, qPCR may underestimate phage activity due to its inability to distinguish viable from nonviable cells. In this study, we evaluated the suitability of propidium monoazide (PMA), a microbial membrane-impermeable dye that inhibits amplification of extracellular DNA and DNA within dead or membrane-compromised cells as a means of using qPCR to identify only intact *E. coli* cells that survive phage exposure. *Escherichia coli* O157:H7 strain R508N and 4 phages (T5-like, T1-like, T4-like, and O1-like) were studied. Results compared PMA-qPCR and direct plating and confirmed that PMA could successfully inhibit amplification of DNA from compromised/damaged cells *E. coli* O157:H7. Compared to PMA-qPCR, direct plating overestimated (*P * < 0.01) phage efficacy as cell surface-attached phage particles lysed *E. coli* O157:H7 during the plating process. Treatment of samples with PMA in combination with qPCR can therefore be considered beneficial when assessing the efficacy of bacteriophage for biocontrol of *E. coli* O157:H7.

## 1. Introduction

Enterohemorrhagic* Escherichia coli* (EHEC) O157:H7 has been recognized as a major food safety concern due to its low infectious dose and severity of disease [1]. Over the last decade, despite the best efforts of the food industry, a number of* E. coli* O157:H7 outbreaks associated with the consumption of contaminated food including spinach [2], ready-to-eat salad, [3] and ground beef [4] have occurred. These outbreaks stress the importance of developing new, more potent strategies to mitigate this important pathogen. Recently, the possibility of using bacteriophages to reduce the concentration of* E. coli *O157:H7 in various foods has gained attention [5–11].

Bacteriophages (phages) are viruses that are natural predators of bacteria, specifically targeting bacterial species or strains, rather than human, plant, or animal cells. The current technique used to determine whether these phages are effective is based on enumeration of bacterial cells by culture on selective media following phage exposure. One problem with culture-based techniques is that EHEC and some other human pathogens (e.g.,* Salmonella enteritidis*,* Vibrio cholerae*,* Legionella pneumophila, Listeria monocytogenes,* and* Campylobacter jejuni*) may enter a viable but nonculturable (VBNC) physiological state in which they do not grow on media [12]. The VBNC state can be induced by stressful conditions such as fluctuating temperatures and oxygen levels [13] and lead to underestimation of the number of viable pathogens during plate counts. Another problem for traditional plating techniques is that surviving bacteria can be killed by cell surface-attached phage particles, during the incubation required for plating, leading to an overestimation of phage efficacy.

Molecular tools, such as qPCR, could solve the VBNC problem but may also overestimate living cell densities due to amplification of DNA from nonviable cells and extracellular DNA within samples [14]. Selection of DNA-binding dyes to eliminate DNA from extracellular cells before cell lysis treatment for PCR could increase accuracy of the method. Propidium monoazide (PMA) covalently binds to DNA bases every 4-5 nucleotides upon exposure to light, forming a carbon-nitrogen bond that inhibits further PCR amplification [15, 16]. PMA is excluded from cells with intact cytoplasmic membranes, thus inactivating extracellular DNA or DNA contained in dead cells, enabling PCR to only amplify DNA within intact cells [17].

PMA-qPCR has been used for enumeration of viable pathogens in water [18, 19], human feces, wastewater influent and effluent [20–22], municipal sewage sludge and biosolids [16], cooked ham [23], dairy products [24], vegetable wash water [25], milk [26], vegetables [27–30], and chemically treated food-contact surfaces [31]. However, the potential of PMA-qPCR to more accurately predict phage efficacy has not been assessed. Accordingly, the objective of this study was to evaluate the usefulness of the optimized PMA-qPCR method to quantify survival of* E. coli* O157:H7 cells in broth inoculated with four types of phage as compared to the traditional plate counting technique.

## 2. Materials and Methods

### 2.1. Bacterial Strains

*E. coli* O157:H7 nalidixic acid resistant strain R508N (bovine origin) was used in this study and a single colony was inoculated into 10 mL of tryptic soy broth (TSB; Difco, Becton Dickinson, Sparks, MD, USA) containing 50 mg/L nalidixic acid (Sigma Chemical Co., Oakville, ON, Canada) and incubated for 18 h at 37°C.* E. coli* O157:H7 in cultures was serially diluted and then enumerated by direct plating onto tryptic soy agar supplemented with 50 mg/L nalidixic acid (TSA-nal; Dalynn Biologicals, Calgary, AB, Canada) after incubation for 18 h at 37°C. Only plates containing 30 to 300 colonies were used for enumeration.

### 2.2. Bacteriophages Selection and Propagation

Four* E. coli* O157:H7 bacteriophages having possibly different mechanisms for degradation of bacterial host DNA were used: T5-like (T5; vB_EcoS_AKFV33) [32], T1-like (T1; vB_EcoS_AHP24) [33] of* Siphoviridae*, T4-like (T4; V7, Public Health Agency of Canada, Laboratory for Foodborne Zoonoses) [34], and O1-like (O1; vB_EcoM_AHP24, unpublished data, Niu et al.) of* Myoviridae*. Stock solutions were prepared for each phage by combining 100 *μ*L of phage suspension (containing 10^8^ to 10^11^ PFU/mL) with 6 mL of a mid-log-phase culture of* E. coli* O157:H7 R508 (OD_600_ = 0.5 to 0.55) followed by incubation of the mixture for 15 min at 37°C to allow phage to attach to the host. An additional 200 mL of TSB amended with MgSO_4_ at 10 mmol/mL (mTSB) was added to the mixture and further incubated for 5 to 10 h at 37°C with shaking (190 rpm), the mixture was then centrifuged at 3700 ×g for 40 min and filtered through a 0.2 *μ*m pore size SFCA serum filter (Nalgene, Rochester, NY, USA). Titres of phages in the stock filtrates were then determined using a soft agar (0.6%) plaque assay [35]. Stock preparations of T5, T1, T4, and O1 contained 4.45 × 10^10^, 2.18 × 10^9^, 3.50 × 10^8^, and 1.26 × 10^10^ PFU/mL, respectively, and were stored at 4°C.

### 2.3. PMA Protocol Optimization

Propidium monoazide (PMA, phenanthridium, 3-amino-8-azido-5-[3-(diethylmethylammonio) propyl]-6-phenyl dichloride, Biotum Inc., Hayward, CA, USA) was stored as a 20 mM stock solution at −20°C in the dark.

To determine the appropriate PMA concentration, differing amounts of PMA (50, 100, or 200 *μ*M) were added to 2 mL microcentrifuge tubes (Axygen, Inc. Union, CA, USA) containing 1.68 × 10^9^, 1.68 × 10^7^, 1.68 × 10^5^, or 1.68 × 10^3^ CFU of heat lysed (99°C, 5 min)* E. coli* O157:H7 R508N. After incubation at 20°C in the dark for 5 min with shaking (200 rpm), the PMA-*E. coli* mixture was placed on ice at a distance of 20 cm from two 500-W halogen lamps for 5, 10, or 20 min. After light exposure, excess PMA was removed by centrifugation (10,000 ×g, 5 min) and DNA was extracted as described in Section 2.4.

To assess if PMA was entering intact cells, 1.68 × 10^5^ and 1.68 × 10^3^ CFU of R508N cells were mixed with 50, 100, 200, or 300 *μ*M of PMA. After 5 min of shaking (200 rpm) at 20°C in the dark, solutions were exposed to light for 5, 10, or 20 min as described above. Positive controls containing live cells but no PMA were also included for each light exposure time. After centrifugation at 10,000 ×g for 5 min, the cell pellets were used for DNA extraction. A 1 mL aliquot of viable* E. coli* O157:H7 cells (PMA-free) was directly plated onto TSA-nal plates at the same time.

To assess the ability of PMA to distinguish intact versus compromised/damaged cells, viable (3.3 × 10^4^ CFU/mL, 5.5 × 10^4^ CFU/mL, 8.0 × 10^4^ CFU/mL, or 1.1 × 10^5^ CFU/mL) and heat lysed (1.1 × 10^5^ CFU/mL)* E. coli* O157:H7 R508N cells were mixed to achieve relative proportions (CFU_live_/CFU_dead_) of 0%, 30%, 50%, 70%, and 100%. Both live and dead fractions were then exposed to PMA as described above. An aliquot (1 mL) of PMA and PMA-free viable and compromised/damaged* E. coli* O157:H7 cells were plated on TSA-nal plates at the same time.

### 2.4. DNA Extraction

Genomic DNA was extracted using the NucleoSpin Tissue kit for cultures (MACHEREY-NAGEL, Düren, Germany) following the manufacturer's instructions, except that a prelysis was included where cells were mixed with 25 *μ*L proteinase K for 1 h at 56°C with agitation at 300 rpm, prior to the standard lysis step. DNA was quantified using Pico Green (Molecular Probes Inc., Eugene, OR, USA) and a NanoDrop 2000 (Thermo Fisher Scientific Inc., Wilmington, DE, USA) using Genomic DNA from* E. coli* O157:H7 as a standard. Results obtained with the NanoDrop 2000 spectrophotometer were compared with band intensities of high molecular weight genomic DNA visualized on ethidium bromide strained 1% agarose gels.

### 2.5. Real-Time PCR

Real-time PCR for detection of viable bacteria was performed in duplicate using an ABI 7500 Fast Real-Time PCR System (Applied Biosystems) and final volume of 20 *μ*L, containing 0.2 mM of forward and reverse primers, 10 *μ*L of 2 × Fast SYBR Green Master Mix (Applied Biosystems, CA, USA) and 2 *μ*L of prepared DNA template. Primer sequences were (F) AGG GGT TGT ATG CTC GTT GT and (R) TGG AAC ACC TTC AAC TTG CTC T [36], specifically targeting a 121-bp segment of the* wzx* gene encoding for O antigen flippase of EHEC O157:H7. Cycling conditions included an initial activation step at 95°C for 20 s, followed by 40 cycles of denaturation at 95°C for 3 s, and annealing temperatures at 60°C for 30 s. After 40 PCR cycles, melting curves were generated at steps of 95°C for 15 s, 60°C for 1 min, followed by 95°C for 30 s, and 60°C for 15 s. Threshold cycle (*C*_*t*_) values were automatically generated by the 7500 Fast Real-Time PCR software. In all cases, negative and positive controls of amplification were included using 2 *μ*L nuclease free water (Applied Biosystems, CA, USA) or known genomic DNA isolated from R508N, respectively.

To quantify the number of* E. coli* O157:H7 cells in samples, a standard curve was prepared from each plate in triplicate for each DNA concentration. Standard curves were generated by amplifying a DNA dilution series of a known number of* E. coli* O157:H7 cells.

### 2.6. Comparison of Real-Time PCR and Direct Plating

To assess the differences between real-time PCR and traditional plating, pure cultures of* E. coli* O157:H7 R508N were incubated at 37°C for 4 h and a 1 mL subsample was withdrawn after 0, 2, and 4 h, respectively. DNA was extracted and real-time PCR was conducted as described in sections 2.4 and 2.5 with 100 *μ*L of a serial diluted subsample being plated onto TSA-nal plates at the same time. Plates were incubated at 37°C for 18–24 h.

### 2.7. Analysis of Phage-Treated Samples

Susceptibility of* E. coli* O157:H7 strain R508N to the four phages was determined by mixing 5 mL of 100 fold-diluted overnight culture (1.2 × 10^7^ CFU/mL) with 10 mL of phages T5 (5.07 × 10^10^ PFU/mL), T1 (3.09 × 10^9^ PFU/mL), O1 (2.55 × 10^10^ PFU/mL), or T4 (6.65 × 10^8^ PFU/mL) in 50 mL Falcon tubes. A negative control consisting of culture inoculated into mTSB without phage was also included. Samples were incubated at 37°C for 4 h. Following incubation, a 1 mL aliquot was dispensed into a 2 mL microcentrifuge tube and centrifuged at 16,000 ×g for 10 min at 4°C. Supernatant was carefully aspirated and the pellets were resuspended in 50 *μ*L phosphate buffer solution (PBS). Samples were then treated with the optimized PMA protocol as described above. Phages from pellets and supernatants were enumerated separately but simultaneously using the soft agar overlay technique described above.

### 2.8. Microplate Phage Virulence Assay

Susceptibilities of* E. coli* O157:H7 R508N to the four phages were determined by a 10-fold serial dilution of 20 *μ*L of phages T5, T1, T4, or O1 at titers of 1.1 × 10^9^, 6.9 × 10^9^, 1.3 × 10^9^, or 4.9 × 10^10^ PFU/mL, respectively, in 180 *μ*L volumes of mTSB in 96-well microplates. Duplicate wells of each phage dilution were then inoculated with 20 *μ*L of the test bacterial culture and the plates were incubated at 37°C for 5 h. Control microplate wells contained mTSB diluent inoculated with bacteria only, while wells containing only phage were used as negative control. Phage plus the host strain R508 was used as positive control. After incubation, the wells were visually inspected for turbidity due to bacterial growth and the highest dilution of phages resulting in lysis (no discernable turbidity) of R508N was recorded.

### 2.9. Statistical Analysis

Colony forming units as determined by qPCR and direct plating were log-transformed and a mixed model analysis of variance was conducted to examine the differences between qPCR and direct plating as well as the ability of PMA to distinguish dead versus live cells using SAS 9.3 (SAS Institute Inc., Cary, NC). Significance was declared at *P* < 0.05.

## 3. Results and Discussion

### 3.1. Development of PMA Real-Time PCR Assay for Quantification of Live and Compromised/Damaged* E. coli* O157:H7 Cells

**Figure 1:**
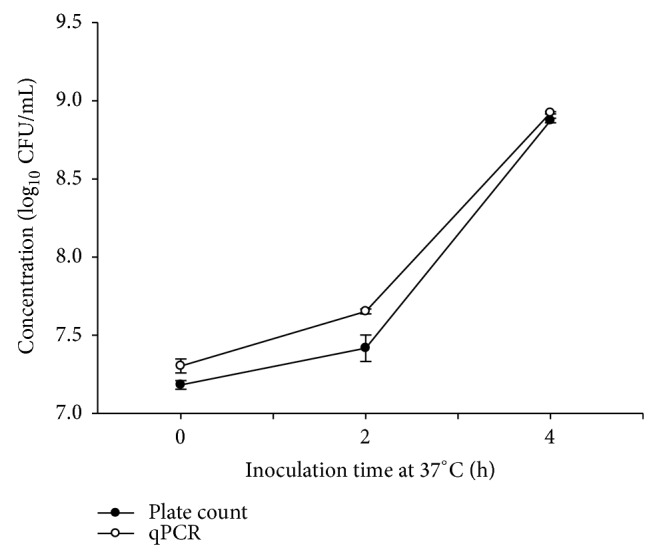
Comparison of accuracy of direct plating and qPCR for pure cultures of viable* E. coli* O157:H7 R508N cells. The difference between direct plating and qPCR was not significant (*P* > 0.05) and the correlation coefficient was 0.94. Each point presents the mean ± standard deviation of two independent replicates.

The use of PMA to prevent amplification of target DNA from compromised/damaged cells in real-time PCR assays has been applied successfully for a variety of organisms and matrices [26, 27, 29–31, 37–39]. However, PMA-qPCR assays require optimization for each bacterial strain and matrix condition of interest. Therefore, we first identified an appropriate qPCR method for the detection of* E. coli* O157:H7 R508N. Through all experiments, the efficiency of the real-time PCR with Fast SYBR Green was between 91 and 100% and the correlation coefficient was between 0.99 and 1.00. In order to minimise false positive results, a qPCR reaction was considered positive only if the* C*_*t*_ value was < 35. The* wzx* gene coding for O antigen flippase has been extensively used as a genetic target for detection or quantification of* E. coli* O157:H7 [36, 40, 41] as only one copy of this gene is present in the genome and standard curves for converting PCR copy number to CFU using this genetic target were available. When pure cultures of viable* E. coli* O157:H7 R508N were assessed, the correlation coefficient between qPCR and direct plating was 0.94 and the number of cells determined by qPCR did not differ from direct plating (*P* > 0.05, Figure 1), likely as amplification of DNA from compromised/damaged cells was minimal.

**Figure 2:**
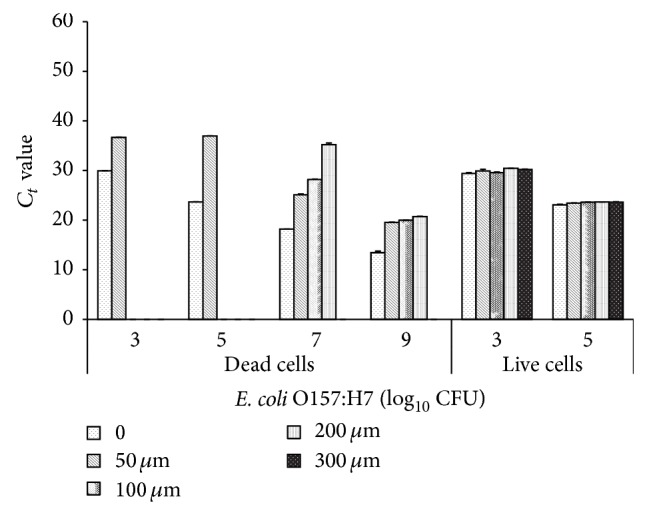
Effect of propidium monoazide (PMA) concentration on detection of live or compromised/damaged* E. coli* O157:H7 R508N cells by qPCR. Live or heat-killed cells were incubated with increasing concentrations of PMA. 300 *μ*M PMA was not included in incubations with dead cells. Amplification of DNA was not detected for 100 and 200 *μ*M PMA for 3 and 5 log_10_CFU/mL compromised/damaged cells. Each bar presents the mean ± standard deviation for two independent replicates. *C*_*t*_ (threshold cycle) > 35, no signal or only a weak signal was detected.

Next, we defined optimal PMA concentrations and appropriate incubation times to differentiate viable from nonviable* E. coli* O157:H7 cells after exposure to phage. To accurately quantify live cells within a mixture with dead cells, PMA should completely inhibit amplification of DNA from compromised/damaged cells without altering the ability to amplify DNA of live cells. Real-time PCR amplification of nucleic acids from compromised/damaged cells of* E. coli* O157:H7 R508N at concentration of 5 log_10_ CFU/mL or less was completely inhibited after treatment with PMA at 50 and 100 *μ*M, as indicated by an increase in the *C*_*t*_ values (Figure 2), as *C*_*t*_ values are inversely proportional to DNA amplification. When DNA extracted from 9 log_10_ CFU of heat-killed R508N cells was treated with 200 *μ*M of PMA, DNA equivalent to about 5 log_10_ CFU of cells remained amplifiable, whereas when DNA was extracted from 7 log_10_ CFU of heat-killed cells with 200 *μ*M PMA no amplification occurred (*C*_*t*_ > 35, no signal or only a weak signal was detected, Figure 2). Consequently, inhibition of qPCR by PMA was incomplete when DNA was extracted from more than 7 log_10_ CFU of compromised/damaged* E. coli* O157:H7 cells (Figure 2). At higher concentrations of compromised/damaged cells, sample turbidity may have affected the efficiency of PMA-qPCR by reducing the light intensity required for photoactivation of the dye. Photoactivation is essential both for binding of the intercalated dye to DNA and for hydrolytic destruction of excess dye that has not entered cells [42]. Accordingly, Wagner et al. [43] and van Frankenhuyzen et al. [16] found PMA to be ineffective at binding extracellular DNA in sewage sludge matrices and suggested that the turbidity of samples was responsible for this failure. Also, PMA might not be completely activated due to the unknown light spectrum that halogen lamp emitted.

To assess if PMA will combine with the DNA of intact cells, viable* E. coli* O157:H7 R508N cells were treated with varying PMA concentrations (50 to 300 *μ*M) over a range of exposure times (5 to 20 min). Amplification after exposure to light for 10 min was similar to controls that were not exposed to PMA, regardless of PMA concentration (Figure 2). Furthermore, intact cells exposed to light for 10 min in the presence of 300 *μ*M PMA amplified to the same extent as PMA negative controls, demonstrating that PMA did not interfere with the amplification of DNA in live cells, even at this high concentration (Figure 2). Based on these findings, further experiments were conducted by exposing samples to light for 10 min in the presence of 100 *μ*M of PMA.

**Figure 3:**
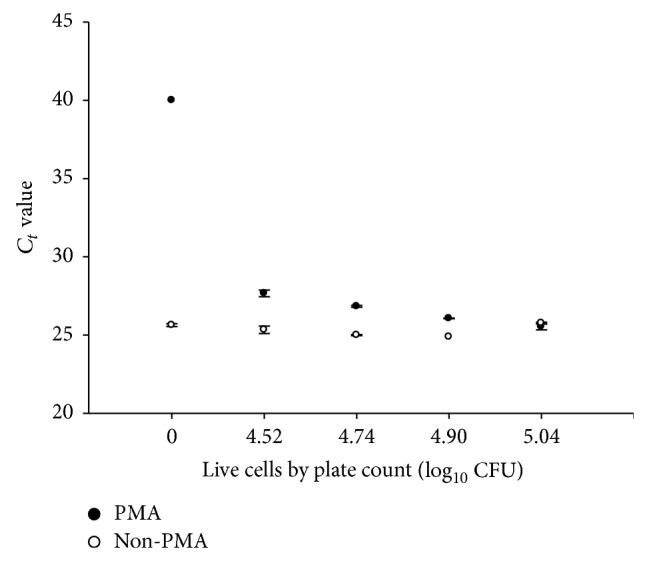
Interference of compromised/damaged cells of* E. coli* O157:H7 during quantification of intact cells by real-time PCR.* E. coli* O157:H7 cells numbers were measured by plate count (culturable), real-time PCR (total), and PMA-qPCR (live) methods. Each point presents the mean ± standard deviation for two independent replicates. *C*_*t*_ (threshold cycle) > 35, no signal or only a weak signal was detected.

To accurately quantify live cells within a mixture of compromised/damaged cells, PMA should completely inhibit amplification of DNA from damaged cells without altering the ability to amplify DNA from intact cells. As PMA-qPCR appeared to be effective for enumeration of intact* E. coli* O157:H7 cells if mixtures contained less than 7 log_10_ CFU of dead cells, another experiment was designed to address the sensitivities of the detection strategies. The intent was to see how well different ratios of live and compromised/damaged cells would impact qPCR signal intensities. Untreated original culture* E. coli* O157:H7 cells were mixed with heat-killed cells (at a concentration of 1.1 × 10^5^ CFU/mL of R508N) in defined ratios with relative proportions (CFU_live_/CFU_dead_) of 0%, 30%, 50%, 70%, and 100%, after PMA or without PMA treatment. Genomic* E. coli* O157:H7 R508N DNA from these mixtures was then quantified by qPCR (Table 1). Without PMA treatment, *C*_*t*_ values decreased as the proportion of dead R508N cells increased in the mixture, demonstrating the ability of DNA from compromised/damaged cells to be readily amplified when mixed with DNA from live cells. After PMA treatment of a mixture of live and compromised/damaged* E. coli* O157:H7 cells, the *C*_*t*_ values increased as the number of live cells in the mixture decreased, demonstrating that PMA inhibited the amplification of genomic DNA from compromised/damaged cells (Figure 3). *C*_*t*_ values obtained from mixture of intact and compromised/damaged cells treated with PMA were comparable to *C*_*t*_ values obtained from controls that contained a similar number of intact* E. coli* O157:H7 not treated with PMA. A previous study showed that *C*_*t*_ values were not influenced by the presence of DNA from heat-killed cells if samples were treated with 200 *μ*M PMA [16]. In our study, we found treatment of live-compromised/damaged cell mixtures to be more efficacious with 100 *μ*M of PMA, a concentration that was two- and tenfold higher than that used by Nocker et al. [15] and Contreras et al. [44], respectively. The higher 100 *μ*M concentration of the present study may have been necessary to compensate for cellular debris in the mixtures interfering with light penetration and reducing the binding efficiency of PMA to DNA.

### 3.2. Use of PMA-qPCR for Enumeration of Survival* E. coli* O157:H7 after Phage Treatment

**Figure 4:**
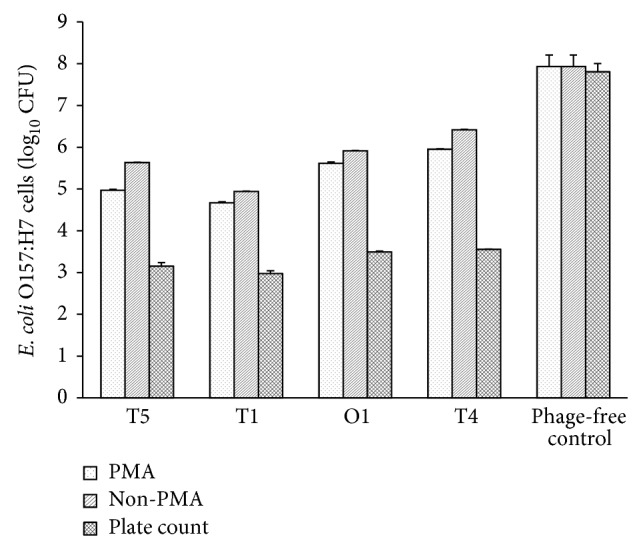
Selective quantification of survival* E. coli* O157:H7 R508N in bacteriophage treated samples.* E. coli* O157:H7 numbers were estimated by plate count (culturable), real-time PCR non-PMA (intact + compromised/damaged), and PMA-qPCR (intact only) methods. Samples were incubated at 37°C for 4 h. The mean ± standard deviation after exposure to four phages at each sampling time was compiled from two independent replicates.

PMA-qPCR was applied to bacterial suspensions exposed to T5, T1, T4, or O1 phages and incubated at 37°C for 4 h. Quantification of* E. coli* O157:H7 derived from qPCR determination of PMA-treated and nontreated samples is shown in Figure 4. After 4 h lysis with T5, T1, O1, or T4 phages, survival of* E. coli* O157:H7 cells in PMA-treated samples was estimated to be lower than non-PMA treated samples (*P* < 0.01) but higher (*P* < 0.01) than plate counts. Use of PMA readily allowed live and dead* E. coli* O157:H7 cells to be differentiated after phage treatment. When* E. coli* O157:H7 cell suspensions treated with a phage were enumerated by plate count, cell numbers were substantially lower (*P* < 0.01) compared to estimates by PMA-qPCR (Figure 4), suggesting that either some cells in the mixture had reverted to a VBNC state or residual phages were lysing* E. coli* O157:H7 during the plating process. Cells that are in the VBNC state are still metabolically active but do not undergo cellular division on conventional media and, therefore, will not produce colonies [12, 45]. Consequently, phage efficacy was overestimated by plate counts.

Alternatively, surface-attached phage particles could be another explanation for the overestimation of the efficacy of phage against* E. coli* O157:H7 by plating. Results from qPCR of pure cultures of live* E. coli* O157:H7 R508N cells were correlated (*r* = 0.94) with direct plating (Figure 1), but numbers of* E. coli* O157:H7 cells remaining after phage treatment were estimated to be higher (*P* < 0.01) by PMA-qPCR as compared to direct plating. To understand this apparent contradiction, phages from pellets and supernatants were enumerated separately using the soft agar overlay technique. After centrifugation of phage-treated samples, about 40% of residual phages were associated with the pellet and 60% were associated with the supernatant (data not shown). Based on these results, we hypothesize that lowered plate counts as compared to PMA-qPCR were due to lysis of* E. coli* O157:H7 by surface-attached phages during plate enumeration. Such an outcome would overestimate the efficacy of phage at killing* E. coli* O157:H7. After treatment with T5, T1, T4, or O1 phages, concentration of* E. coli* O157:H7 by PMA-qPCR was lower (*P* < 0.01) than that determined by qPCR without PMA, which detected DNA from both live and compromised/damaged cells (Figure 4). Consequently, PMA-qPCR could successfully solve the problem of overestimation of phage efficacy against* E. coli* O157:H7, which mostly arose as a result of the lyses of surviving* E. coli* O157:H7 by surface-attached phage particles during the plating process. Estimation of phage efficacy using the direct cultural plating is also considerably more time consuming requiring more than 48 h for bacteria to be grown and enumerated, whereas PMA-qPCR can estimate phage efficacy in as little as 5 h.

Additionally,* E. coli* O157:H7 strain R508N was >10^2^ times more sensitive to T5 and T1 than to phages O1 and T4 (Table 2), indicating the former were more effective in killing bacteria than the latter. This outcome was reflected by the results of using PMA-qPCR to assess phage efficacy.

### 3.3. Fate of Host DNA after Phage Infection

The process of bacteriophage multiplication in bacteria involves a number of mechanisms by which the host metabolism is modified to direct the synthesis of phage-specific macromolecules [46]. However, phage-induced degradation of host DNA by different phages remains obscure. From Figure 4, the estimated concentration of* E. coli* O157:H7 cells using qPCR without PMA, which would theoretically include both intact and compromised/damaged cells, was 1 to 2 log_10_ CFU lower than the original inoculant (log_10_ CFU), likely as some DNA from compromised/damaged cells was thoroughly degraded by phage nucleases and not detected by qPCR. Based on non-PMA values, degradation of host DNA was greater for T1 than T5 (*P* < 0.01) and greater for T5 than O1 or T4 (*P* < 0.01). The difference between non-PMA versus PMA for detection of* E. coli* O157:H7 cells also varied among phages, possibly due to the differing mechanisms used by these phages to degrade host DNA [47–49]. Phage-induced degradation of host DNA can lead to host cell death in the absence of phage replication. Phage T5 degrades host DNA in a rapid and complete manner [50–52]. McCorquodale [49] suggested that the protein specified by gene Al of phage T5 is responsible for the degradation of host DNA to acid-soluble products, a conversion that occurred within the first minute after infection at 37°C.

Phage nucleases, especially endonucleases, play an important role in the degradation of* E. coli* DNA [47]. Parson and Snustad [48] showed that host DNA degradation after infection of* E. coli* with phage T4 depended on the presence of T4 endonuclease IV. Among the four different types of phages used in the present study, proteins involved in lytic-specific DNA replication and repair are unevenly distributed. Roberts et al. [53] showed that there were three endonucleases involved in host DNA degradation in phage T1. Also, three endonucleases (I-TevI, I-TevII, and I-TevIII) were found in phage T4 genome [54]. Whichard et al. [55] showed that the phage O1 genome contained six copies of sequences homologous to homing endonucleases. Compared to T4 and T5, T1 and O1 phages have been less studied with respect to their biochemical physiology and the nature of their DNA endonucleases. This study suggests that degree of degradation of host bacterial DNA varies among phages and that this may have also contributed to the variation in DNA detection observed between qPCR and PMA-qPCR.

## 4. Conclusions

To our knowledge, this study is the first to combine PMA with real-time PCR for evaluating the efficacy of phages as a method of pathogen biocontrol. When applied to* E. coli* O157:H7 and compared to traditional plating, PMA-qPCR was rapid, effective, and consistently estimated higher numbers of intact* E. coli* O157:H7 than plate counts after exposure to phage. The PMA-qPCR method could potentially be used to monitor survival of* E. coli* O157:H7 after treatment with phages, particularly in cases where a rapid result is needed as only 5 h was required as compared to 48 h with standard plating. Data from these studies suggest that PMA-qPCR may yield a more accurate estimation of intact* E. coli* O157:H7 cells than traditional culture methods, providing that the number of compromised/damaged cells does not exceed 7 log_10_ CFU/mL and thus may be an improved method for evaluating the efficacy of phages at inactivating EHEC. To further validate the qPCR method, PMA should be evaluated for other pathogens of interest, such as* Salmonella *and* Campylobacter*.

## Figures and Tables

**Table 1:** Assessment of the extent to which proportion of compromised/damaged *E. coli* O157:H7 R508N cells interfere with quantification of intact cells as estimated by qPCR.

Live/dead cells (%)	0	30	50	70	100
Live (log_10_⁡CFU)	0	4.5 ± 0.02	4.7 ± 0.02	4.9 ± 0.03	5.0 ± 0.03
Compromised/damaged (log_10_⁡CFU)	5.0 ± 0.0	5.0 ± 0.03	5.0 ± 0.03	5.0 ± 0.03	0
PMA-qPCR					
*C*_*t*_	NA	27.7 ± 0.21	26.8 ± 0.06	26.1 ± 0.02	25.5 ± 0.2
Concentration	NA	4.4 ± 0.04	4.7 ± 0.03	4.9 ± 0.03	5.1 ± 0.04
qPCR without PMA					
*C*_*t*_	25.6 ± 0.09	25.3 ± 0.24	25.0 ± 0.04	24.9 ± 0.05	25.8 ± 0.06
Concentration	5.0 ± 0.04	5.1 ± 0.03	5.2 ± 0.03	5.3 ± 0.03	5.0 ± 0.04

NA: not available—did not amplify.

The mean ± standard deviation of R508N concentration was compiled from two independent replicates.

**Table 2:** Relative susceptibility of *E. coli* O157:H7 to four bacteriophages used in the experiment.

Relative susceptibility (log_10_⁡PFU/mL) to bacteriophages^*^				
*E. coli* O157:H7	T5	T1	O1	T4
R508N	<4	<4	4	6

^*^The lowest titer at which complete lysis of *E. coli* O157:H7 occurred.
